# Supplementary material for: Ecomorphological inferences in early vertebrates: reconstructing Dunkleosteus terrelli (Arthrodira, Placodermi) caudal fin from palaeoecological data
Source: PeerJ. 2017 Dec 6;5:e4081. doi: 10.7717/peerj.4081 (PMC5723140; doi:10.7717/peerj.4081)
Supplement: Data S1 [file peerj-05-4081-s001.pdf]

Supplemental Data S1. Shark species included in the geometric morphometric analyses.

Hexanchiformes: *Chlamydoselachus anguineus*

Echinorhiniformes: *Echinorhinus cookei*

Squaliformes: *Squalus brevirostris, Centrophorus granulosus, Etmopterus pusillus, Centroscymnus owstonii, Somniosus pacificus, Euprotomicrus bispinatus*

Heterodontiformes: *Heterodontus portusjacksoni*

Lamniformes: *Carcharias taurus, Pseudocarcharias kamoharai, Cetorhinus maximus, Carcharodon carcharias*

Orectolobiformes: *Nebrius ferrugineus, Rhincodon typus*

Carcharhiniformes: *Apristurus laurussonii, Atelomycterus marmoratus, Cephaloscyllium pictum, Scyliorhinus stellaris, Gollum attenuatus, Pseudotriakis microdon, Leptocharias smithii, Furgaleus macki, Galeorhinus galeus, Hemitriakis falcata, Carcharhinus perezi, Carcharhinus tilsoni, Isogomphodon oxyrhynchus, Lamiopsis temmincki, Scoliodon laticaudus, Sphyrna lewini*

In red: demersal sharks

In green: squalomorph sharks

In blue: active pelagic sharks

Some taxa were not considered for the geometric morphometrics analysis for different reasons. Squatiniformes were not included for having hypocercal caudal fins. In order to prevent the Pinocchio effect taxa with elongated snout (i.e., Pristiophoriformes and *Mitsukurina owstoni*) or elongated dorsal lobe of the caudal fin (i.e., *Megachasma pelagios*, *Alopias* spp. and *Stegostoma fasciatum*) were also not considered. Finally, some orectolobiform families are not represented for lacking a well-defined ventral tip of the caudal fin, thus making impossible the placement of the Landmark 7.
