# Supplementary material for: Ecomorphological inferences in early vertebrates: reconstructing Dunkleosteus terrelli (Arthrodira, Placodermi) caudal fin from palaeoecological data
Source: PeerJ. 2017 Dec 6;5:e4081. doi: 10.7717/peerj.4081 (PMC5723140; doi:10.7717/peerj.4081)
Supplement: Data S2 [file peerj-05-4081-s002.pdf]

Supplemental Data S2. Total Body Length (TBL) and Upper Jaw Perimeter (UJP) data of extant sharks considered in this study. Data taken from Lowry et al. (2009).

Species	TBL (cm)	UJP (cm)
Carcharodon carcharias	227	40.1
Carcharodon carcharias	262	41.7
Carcharodon carcharias	182.5	26.2
Carcharodon carcharias	170.2	30.1
Carcharodon carcharias	124.5	22.4
Carcharodon carcharias	243.8	41.8
Carcharodon carcharias	379.1	60.3
Carcharodon carcharias	154.9	25.4
Carcharodon carcharias	523.2	99
Carcharodon carcharias	563.2	96.7
Carcharodon carcharias	487.7	80.5
Carcharodon carcharias	237	38.5
Carcharodon carcharias	281.9	46.5
Carcharodon carcharias	134.6	23
Carcharodon carcharias	535.9	90.5
Carcharodon carcharias	594.4	89.2
Carcharodon carcharias	457.2	79.8
Carcharodon carcharias	473.7	91.5
Carcharodon carcharias	553.7	85.5
Carcharodon carcharias	166.37	26.3
Carcharhinus acronotus	107	13.3
Carcharhinus acronotus	111	12.9
Carcharhinus acronotus	94.1	10.5
Carcharhinus acronotus	117.6	13.8
Carcharhinus acronotus	108	13.3
Carcharhinus acronotus	120.4	13.9
Carcharhinus acronotus	129	15.3
Carcharhinus acronotus	129	15.1
Carcharhinus acronotus	87.6	9.1
Carcharhinus acronotus	116	13.3
Carcharhinus acronotus	94	12.6
Carcharhinus acronotus	120.6	13.8
Carcharhinus acronotus	132	16.1
Carcharhinus acronotus	101	11.5
Carcharhinus acronotus	81.3	8.9
Carcharhinus acronotus	88	10.9
Carcharhinus acronotus	114.7	13.5
Carcharhinus acronotus	76	8.5
Carcharhinus acronotus	115	14.1
Carcharhinus acronotus	116	13.4
Carcharhinus brevipinna	213	27.3

Carcharhinus brevipinna	205	25.4
Carcharhinus brevipinna	141	17.9
Carcharhinus brevipinna	202	24
Carcharhinus brevipinna	243.8	28.2
Carcharhinus brevipinna	213	25.5
Carcharhinus brevipinna	160	19.8
Carcharhinus brevipinna	165	24.6
Carcharhinus brevipinna	168	21.5
Carcharhinus brevipinna	174	22.8
Carcharhinus brevipinna	141	21.4
Carcharhinus brevipinna	208	25.5
Carcharhinus falciformis	186	18.8
Carcharhinus falciformis	185	21.7
Carcharhinus falciformis	244	30.2
Carcharhinus falciformis	265	32
Carcharhinus falciformis	92	12
Carcharhinus falciformis	94	14.5
Carcharhinus falciformis	151	19.5
Carcharhinus falciformis	73.5	9.6
Carcharhinus falciformis	119	15.5
Carcharhinus falciformis	86.68	11.2
Carcharhinus leucas	229	37
Carcharhinus leucas	245	42.2
Carcharhinus leucas	278	44.3
Carcharhinus leucas	139.5	23.3
Carcharhinus leucas	173.4	25.8
Carcharhinus leucas	182	27.5
Carcharhinus leucas	254.4	39.8
Carcharhinus leucas	229.5	37.4
Carcharhinus leucas	166.5	25
Carcharhinus leucas	252	39.3
Carcharhinus leucas	254	41
Carcharhinus leucas	278	43.1
Carcharhinus leucas	244	35.4
Carcharhinus leucas	226	33.1
Carcharhinus leucas	266.7	41.2
Carcharhinus leucas	254	41
Carcharhinus leucas	214	28.6
Carcharhinus leucas	284.5	44.2
Carcharhinus leucas	190.5	30
Carcharhinus leucas	222	33.6
Carcharhinus leucas	278	43
Carcharhinus leucas	138	19.9
Carcharhinus limbatus	181.4	25.5
Carcharhinus limbatus	171	26.7
Carcharhinus limbatus	165	26.4

Carcharhinus limbatus	193.5	28.5
Carcharhinus limbatus	78	12.3
Carcharhinus limbatus	158.5	27.2
Carcharhinus limbatus	128.5	18.6
Carcharhinus limbatus	176	26.1
Carcharhinus limbatus	169	24
Carcharhinus limbatus	177.8	26.8
Carcharhinus limbatus	150	24.1
Carcharhinus limbatus	201.9	26.6
Carcharhinus limbatus	170	27.3
Carcharhinus limbatus	176.5	26.8
Carcharhinus limbatus	124	19.5
Carcharhinus limbatus	150	21.5
Carcharhinus limbatus	185.4	26.2
Carcharhinus limbatus	193	28
Carcharhinus limbatus	115	18
Carcharhinus limbatus	136	22.1
Carcharhinus obscurus	320	45.1
Carcharhinus obscurus	300.9	45.7
Carcharhinus obscurus	313	45.9
Carcharhinus obscurus	307	40.2
Carcharhinus obscurus	295	38.1
Carcharhinus obscurus	153	21.2
Carcharhinus obscurus	229	28.3
Carcharhinus obscurus	334	41.2
Carcharhinus obscurus	320	40.7
Carcharhinus obscurus	256.5	34.2
Carcharhinus obscurus	320	44.6
Carcharhinus obscurus	294	38.5
Carcharhinus obscurus	323.9	43.4
Carcharhinus obscurus	182.9	26.3
Carcharhinus obscurus	340.4	46.5
Carcharhinus obscurus	386.1	46.3
Carcharhinus obscurus	310	41.5
Carcharhinus plumbeus	182	27.3
Carcharhinus plumbeus	182	25.6
Carcharhinus plumbeus	165	23.7
Carcharhinus plumbeus	212	27.3
Carcharhinus plumbeus	182	27.3
Carcharhinus plumbeus	186	22.1
Carcharhinus plumbeus	95.6	11.5
Carcharhinus plumbeus	86.8	11
Carcharhinus plumbeus	202	28
Carcharhinus plumbeus	198.12	28.3
Carcharhinus plumbeus	165.1	25.1
Carcharhinus plumbeus	185	27.8

Carcharhinus plumbeus	208	29.6
Carcharhinus plumbeus	196	28.2
Carcharhinus plumbeus	167	23.9
Carcharhinus plumbeus	191	28.4
Carcharhinus plumbeus	192	26.9
Carcharhinus plumbeus	194	29.3
Carcharhinus plumbeus	181	30.7
Carcharhinus plumbeus	153	25.4
Carcharhinus plumbeus	182	26.2
Carcharhinus plumbeus	166	24
Carcharhinus plumbeus	183	29.5
Isurus paucus	156.4	27.8
Isurus paucus	220	35.2
Isurus paucus	265.1	46.7
Isurus paucus	426.7	72.2
Isurus paucus	126.1	23.1
Isurus paucus	272	45.3
Isurus paucus	384.81	64.5
Isurus paucus	228.6	39.4
Isurus paucus	242	43.4
Isurus paucus	251.46	43.8
Isurus paucus	320	57.3
Isurus paucus	220.98	37
Isurus paucus	224.16	37.7
Isurus paucus	285.75	50.5
Isurus paucus	243.84	42
Isurus paucus	262.89	42.3
Isurus paucus	205.7	36.5
Isurus paucus	254	43.2
Isurus paucus	248.48	47.3
Isurus oxyrinchus	259.1	47.2
Isurus oxyrinchus	251	50
Isurus oxyrinchus	198.12	33.9
Isurus oxyrinchus	264.16	44.5
Isurus oxyrinchus	239.4	41.3
Isurus oxyrinchus	303.53	54.3
Isurus oxyrinchus	320.04	56.8
Isurus oxyrinchus	320	58
Isurus oxyrinchus	276.86	51
Isurus oxyrinchus	254	41
Isurus oxyrinchus	228.6	43.2
Isurus oxyrinchus	259.1	45.7
Isurus oxyrinchus	259	47.1
Isurus oxyrinchus	251.5	46
Isurus oxyrinchus	254	43.2
Isurus oxyrinchus	274.3	47.2

Isurus oxyrinchus	264.16	49
Isurus oxyrinchus	190.5	33.5
Isurus oxyrinchus	177	31.8
Isurus oxyrinchus	136	23.9
Isurus oxyrinchus	163	28
Isurus oxyrinchus	181	35.1
Isurus oxyrinchus	246	45.5
Negaprion brevirostris	170	28.3
Negaprion brevirostris	260	37.8
Negaprion brevirostris	243	34.7
Negaprion brevirostris	116	18.6
Negaprion brevirostris	268	40.6
Negaprion brevirostris	232	39.5
Negaprion brevirostris	240	35
Negaprion brevirostris	198	30.6
Negaprion brevirostris	68.5	10.3
Negaprion brevirostris	213.4	39.7
Galeocerdo cuvier	327	54
Galeocerdo cuvier	71.12	10.3
Galeocerdo cuvier	212.3	26.4
Galeocerdo cuvier	235	30.6
Galeocerdo cuvier	335	41
Galeocerdo cuvier	218.3	27.7
Galeocerdo cuvier	300	43
Galeocerdo cuvier	78.8	9.8
Galeocerdo cuvier	219	27.9
Galeocerdo cuvier	185	25.2
Galeocerdo cuvier	337.72	51.2
Galeocerdo cuvier	378.46	52.6
Galeocerdo cuvier	358.14	49.7
Galeocerdo cuvier	208.28	29
Galeocerdo cuvier	186.7	22.1
Galeocerdo cuvier	151	19
Galeocerdo cuvier	182	24.3
Galeocerdo cuvier	95	11.2
Galeocerdo cuvier	138	17.5
Galeocerdo cuvier	358.14	51.9
Galeocerdo cuvier	136	17.7
Galeocerdo cuvier	332	52.1
Sphyrna lewini	246.4	23.5
Sphyrna lewini	277.5	26.5
Sphyrna lewini	230.5	21.8
Sphyrna lewini	277	26.4
Sphyrna lewini	326	29.8
Sphyrna lewini	100	10.6
Sphyrna lewini	161	15.8

Sphyrna lewini	205	20.7
Sphyrna lewini	165	16.8
Sphyrna lewini	250	23.5
Sphyrna lewini	167	16.4
Sphyrna lewini	258	24.4
Sphyrna lewini	301	27.6
Sphyrna lewini	127	13.2
Sphyrna mokarran	414	58.2
Sphyrna mokarran	208	24.5
Sphyrna mokarran	350	41
Sphyrna mokarran	205	22.7
Sphyrna mokarran	314	36.6
Sphyrna mokarran	257	28.4
Sphyrna mokarran	246	25
Sphyrna mokarran	298	32.9
Sphyrna mokarran	319	36.5
Sphyrna mokarran	255	27.8
Sphyrna mokarran	302	31.2
Sphyrna mokarran	300	40.5
Sphyrna mokarran	365	29.5
