# Supplementary material for: Ecomorphological inferences in early vertebrates: reconstructing Dunkleosteus terrelli (Arthrodira, Placodermi) caudal fin from palaeoecological data
Source: PeerJ. 2017 Dec 6;5:e4081. doi: 10.7717/peerj.4081 (PMC5723140; doi:10.7717/peerj.4081)

Supplemental Data S3. GPA (left) and RFTRA (right) superimpositions for the whole group of sharks and for each ecological subgroup. Points denote landmarks and crosses denote landmark centroids.

Total

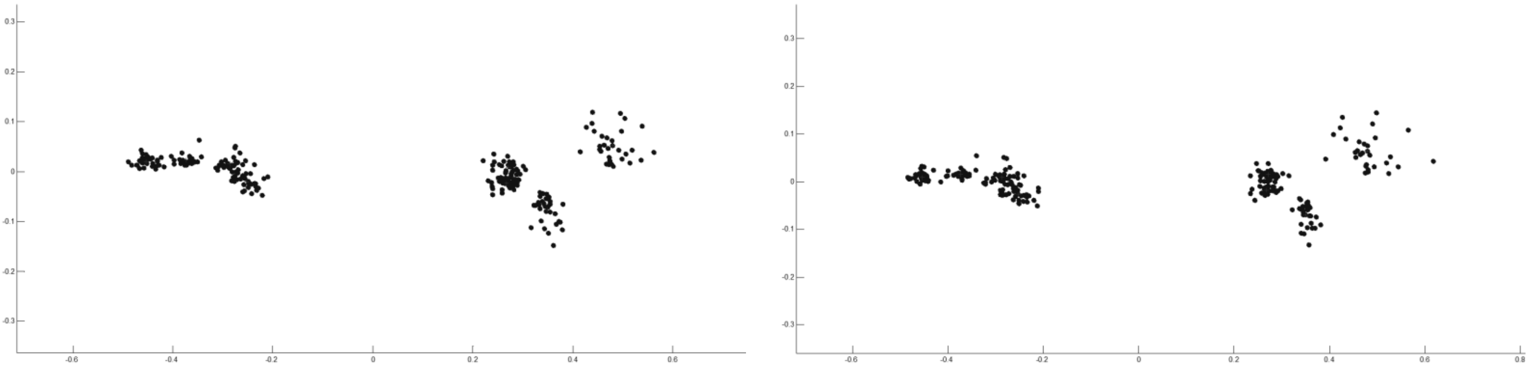

Demersal sharks

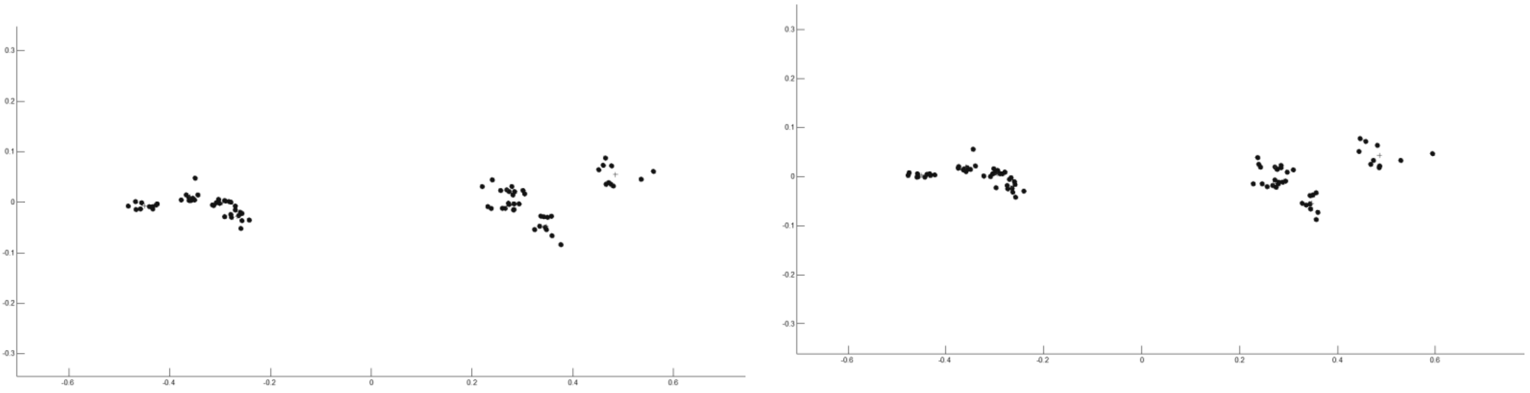

Squalomorph sharks

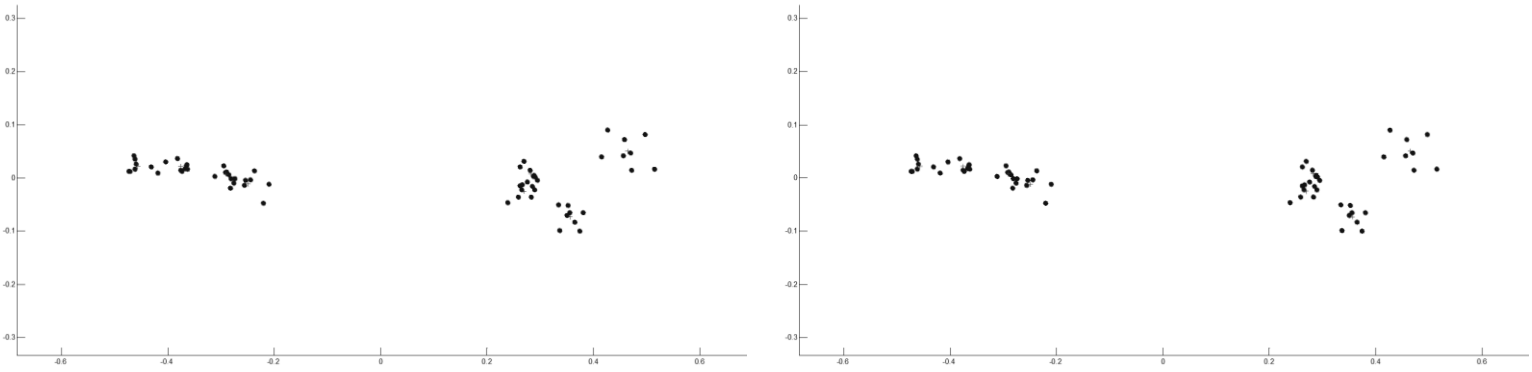

Active pelagic sharks

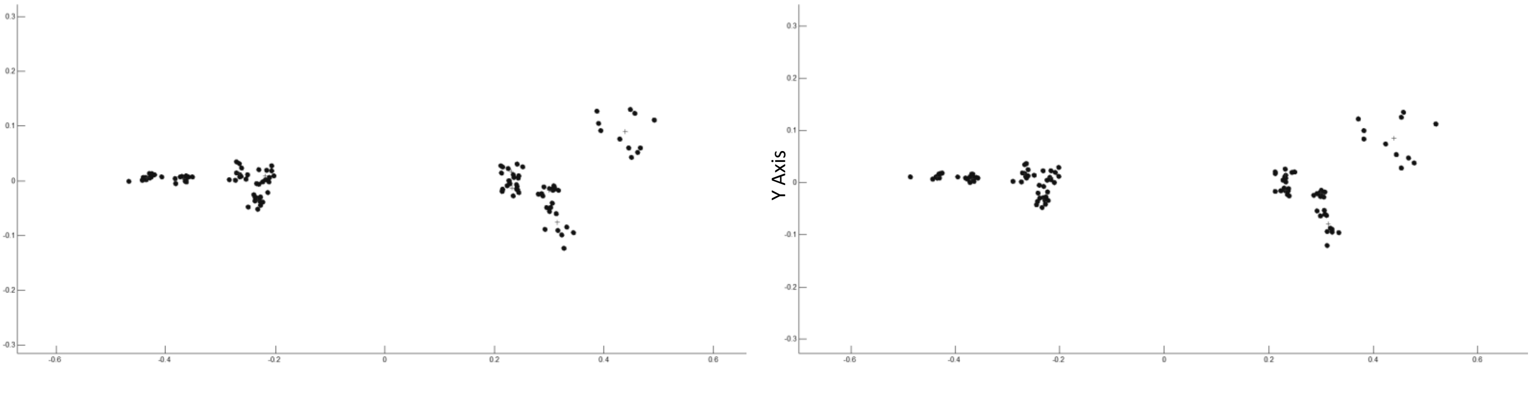

Supplement: Data S3 [file peerj-05-4081-s003.pdf]
