# Supplementary figures and images for: Ecomorphological inferences in early vertebrates: reconstructing Dunkleosteus terrelli (Arthrodira, Placodermi) caudal fin from palaeoecological data
Source: PeerJ. 2017 Dec 6;5:e4081. doi: 10.7717/peerj.4081 (PMC5723140; doi:10.7717/peerj.4081)

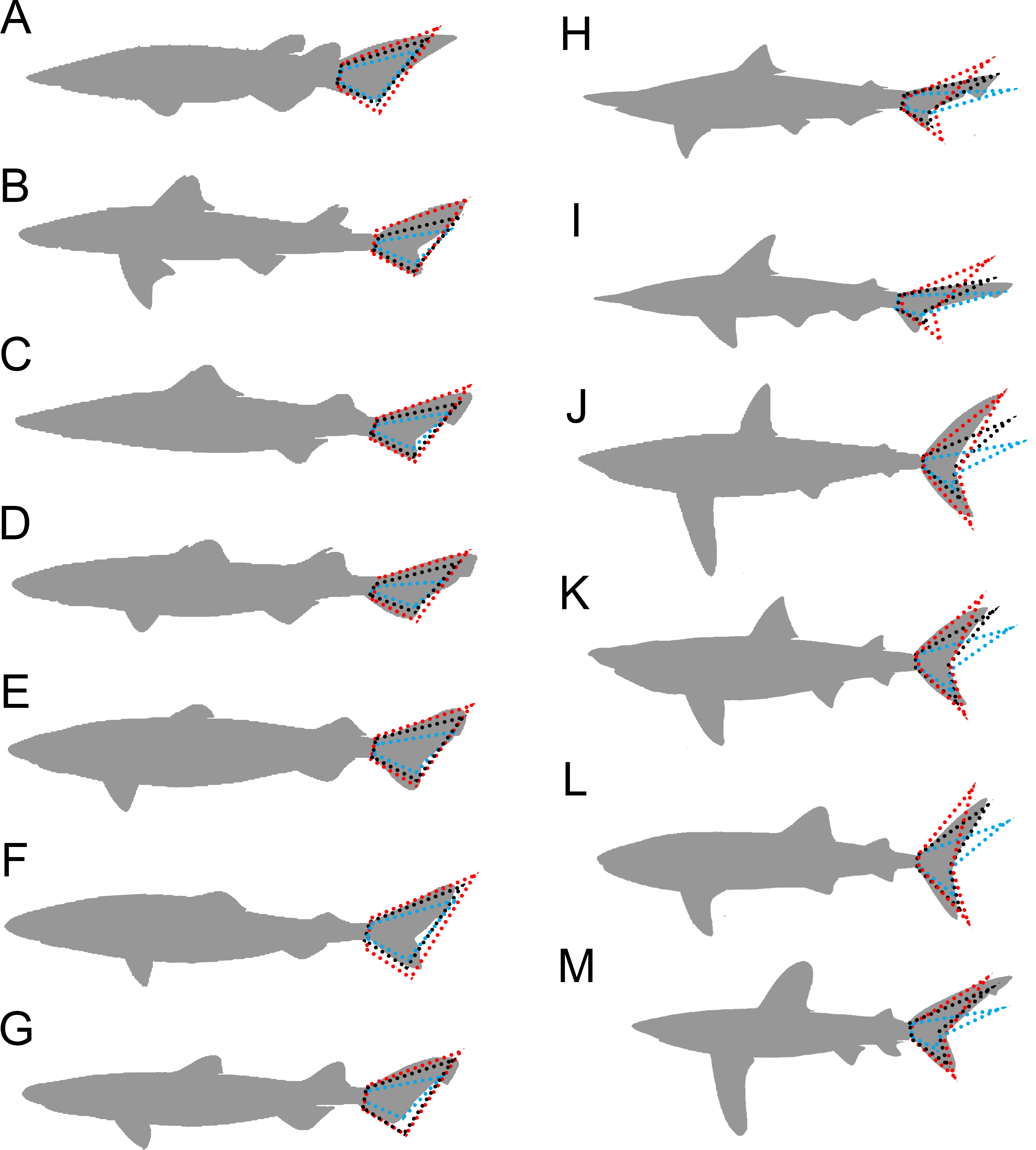

Supplement: Figure S1 — (A) Echinorhinus brucus 310 cm, (B) Squalus cubensis 110 cm, (C) Centrophorus atromarginatus 94 cm, (D) Centroscyllium ritteri 43 cm, (E) Centroscymnus coelolepis 122 cm, (F) Somniosus microcephalus 730 cm, (G) Dalatias licha 182 cm, (H) Rhizoprionodon terraenovae 110 cm, (I) Sphyrna corona 92 cm, (J) Isurus paucus 430 cm, (K) Cetorhinus maximus 1,000 cm, (L) Rhincodon typus 2100 cm, (M) Carcharhinus longimanus 395 cm. Shark outlines modified from Ebert, Fowler & Compagno (2013). [file peerj-05-4081-s004.png]

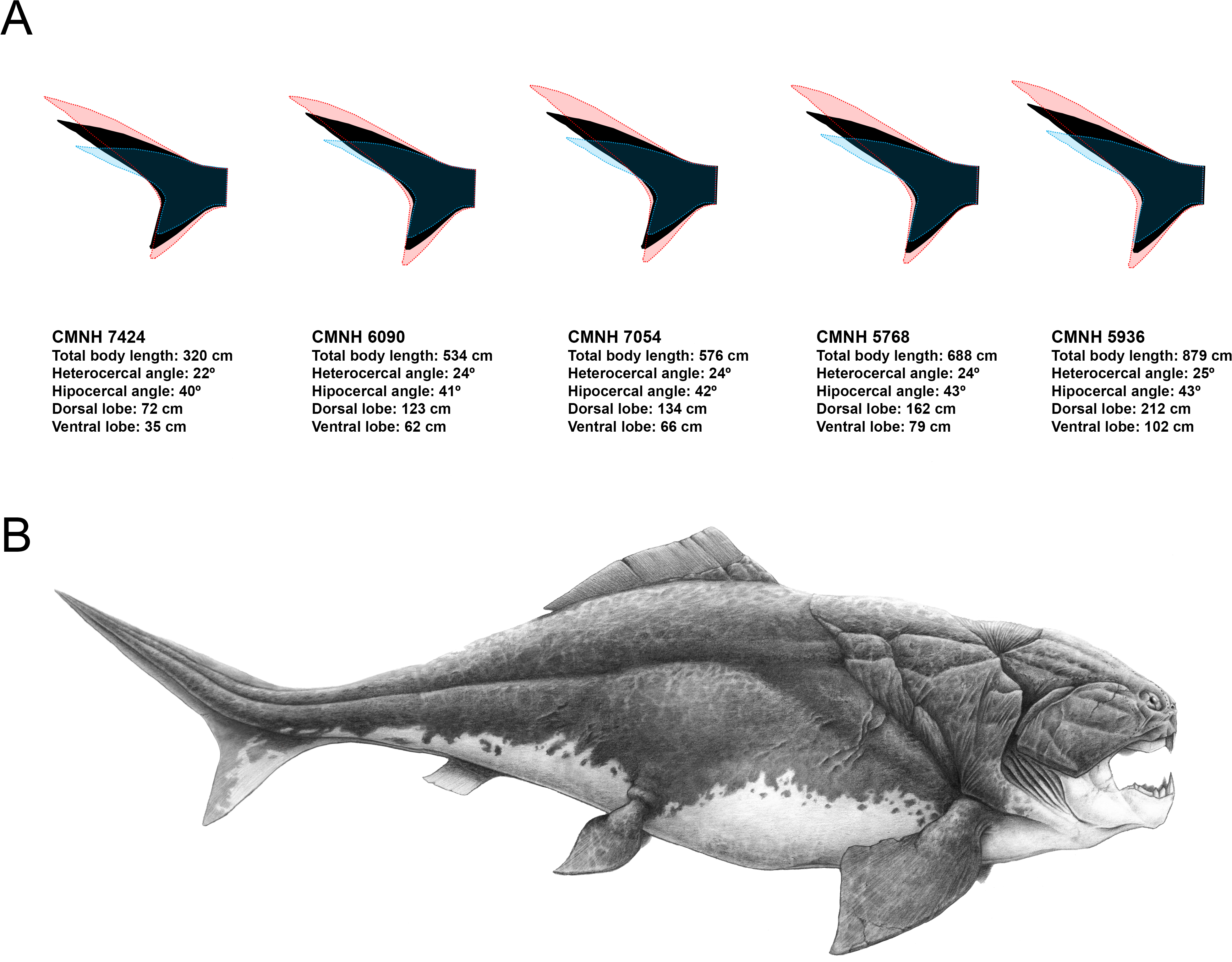

Supplement: Figure S2 — (A) Predicted caudal fin shape of each specimen of D. terrelli (in black), showing the upper and lower 90% individual confidence interval boundaries (in red and blue respectively). (B) Palaeoartistic reconstruction of a 7.33 meters D. terrelli (courtesy of Dr. Hugo Salais, HS Scientific Illustration). [file peerj-05-4081-s005.png]
